# Laparoscopic staging of endometrial cancer: Does it have any impact on survival?

**DOI:** 10.4274/tjod.78466

**Published:** 2015-09-15

**Authors:** Taylan Şenol, Mesut Polat, İlhan Şanverdi, Enis Özkaya, Ateş Karateke

**Affiliations:** 1 Zeynep Kamil Training and Research Hospital, Clinic of Women and Children’s Health, İstanbul, Turkey

**Keywords:** Endometrial cancer, tumor diameter, overall survival, disease-free survival, laparoscopic

## Abstract

**Objective:**

To assess whether laporoscopic approach to endometrial cancer is associated with survival.

**Materials and Methods:**

In total, 158 patients with endometrial cancer underwent staging surgery at a tertiary referral center, 30 of whom underwent laparoscopy, whereas the remainder received treatment with a conventional approach. Survival between groups was analyzed.

**Results:**

The comparison of the groups revealed similar disease-free survival (p=0.791). Histology, cervical, adnexal and serosal involvement were found to be significantly correlated with recurrence in the laparoscopically staged group, whereas CA 125, histology, tumor grade, tumor diameter, cervical involvement, degree of myometrial invasion, adnexal and serosal involvement, and pelvic metastasis were significanly correlated with recurrence in the conventionally managed group.

**Conclusion:**

Laparoscopic approach to endometrial cancer, along with its widely accepted postoperative advantages, has similar disease-free survival but different variables affect recurrence rates.

## PRECIS:

Laparoscopic management of endometrial cancer, along with its widely accepted postoperative advantages, has similar disease-free survival but different variables affect recurrence rates.

## INTRODUCTION

Endometrial carcinomas are the most commonly seen malignancy of the female genital tract in developed countries^(1)^. Most cases present with disease confined to the uterus; a small proportion of patients have more advanced disease^(2)^. The main step in the management of endometrial cancer is surgery, which includes total hysterectomy, bilateral salpingo-oophorectomy, peritoneal washing, and pelvic and paraaortic lymphadenectomy^(3)^. There is still a debate on the indication and extent of lymph node dissection in the surgical management of endometrial cancer^(3,4,5)^. Although there is a consensus for the necessity of complete surgical staging for high-risk endometrial cancers, the requirement for complete surgical staging in low-risk endometrial cancer remains debatable^(6)^. Several factors have been associated with recurrence, survival, or both^(7)^. The laparoscopic approach for treatment of endometrial cancer has only recently been introduced. The effect of endoscopic intervention on survival remains questionable; a recently published study indicated that compared with laparotomy, laparoscopic surgery seemed to be beneficial in women with endometrial cancer in terms of postoperative complications and length of hospital stay. In addition, a review concluded that well-designed randomized controlled studies are needed to assess long-term clinical outcomes(^8)^.

The aim of this study was to assess whether the laporoscopic approach to endometrial cancer is associated with survival.

## MATERIALS AND METHODS

We performed a study in 158 women with endometrial cancer, who were treated at the Department of Gynecologic Oncology of the Zeynep Kamil Maternity and Children’s Training and Research Hospital, between January 2010 to December 2014. Inclusion criteria were endometrial cancer confirmed with pre-operative endometrial sampling, surgical treatment, appropriate data obtained from the database, and regular clinical follow-up. Exclusion criteria were positive endocervical sampling indicating cervical adenocancer, hormone replacement therapy, loss of follow-up, incomplete adjuvant therapy, and incomplete staging surgery. The staging surgery (pelvic-paraaortic lymphadenectomy) was performed based on the frozen section report. At our institution, staging surgery is performed using the following criteria: Grade 1 or 2 endometrioid carcinoma with myometrial invasion > 50%; grade 3 tumors; tumors with cervical extension; clear cell or serous carcinoma; and tumor size >2 cm. Dependent on the result of frozen section report indicating high risk factors for lymph nodal metastases and the surgeon’s clinical evaluation, total staging procedures including pelvic, and eventual paraaortic lymph node dissection were performed. Microscopic evaluation permanent histology, tumor grade, and stage were made in accordance with the World Health Organization and International Federation of Gynecology and Obstetrics (FIGO) classifications. The surgical procedure consisted of an exploratory laparotomy or laparoscopy, peritoneal washings for cytology, hysterectomy, and bilateral salpingo-oophorectomy. After the uterus was removed, it was sent for frozen section (FS) evaluation to determine the depth of myometrial invasion and tumor grade.

The staging surgery procedure (pelvic-paraaortic lymphadenectomy) was performed based on the FS report. The following parameters were recorded for each participant, age, smoking, menopausal status, body mass index, CA 125, systemic disorders, tumor histology, tumor grade, lymphovascular space invasion, tumor diameter, cervical involvement, myometrial invasion, adnexal metastases, positive cytology, serosal involvement, other pelvic metastases, type of surgery, and fertility sparing approach. Thirty women underwent laparoscopy, whereas the remainder were treated using the conventional approach; survival between groups was analyzed.

### Statistical analysis

All data were analyzed using SPSS version 15. All data were summarized using descriptive statistics, correlation analyses were used to show associations, multivariate regression was used to show adjusted associations, ROC analysis was used to calculate predictive value, and sensitivity and specificity. Cox regression was used to determine predictors for survival. Kaplan-Meier survival analyses were used to calculate survival. P<0.05 was accepted to be statistically significant.

## RESULTS

**Figure 1:**
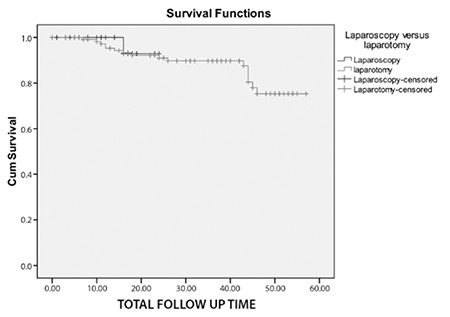
Survival plot of comparison of survival between groups

Demographic and the clinical features of the study population are summarized in Table 1. Comparison of the groups revealed similar disease-free survival (p=0.791, Figure 1). A comparison of clinical and demographic characteristics of the groups is summarized in Table 2. The frequency of histologic subtypes is shown in Table 3. Histology, cervical, adnexal and the serosal involvement were significantly correlated with recurrence in the laparoscopically staged group, whereas CA 125, tumor histology, grade, tumor diameter, cervical involvement, degree of myometrial invasion, adnexal and serosal involvement, and pelvic metastasis were significantly correlated with recurrence in the conventionally managed group. In the Cox regression analyses, none of the variables were found to be significantly associated with disease-free survival in the laparoscopically-or conventionally staged groups. The number of smokers, menopausal status, systemic disorders, tumor grade, lymphovascular space involvement, rate of positive peritoneal cytology, and FIGO stages were all comparable between groups (p>0.05). The number of metastatic pelvic and paraaortic lymph nodes were also similar between groups (p>0.05). There was only 1 recurrence observed in the laparoscopically staged group compared with 16 in the conventionally staged group (p>0.05).

## DISCUSSION

In this study, we analyzed 158 women with endometrial cancer who underwent either laparoscopic surgery or laparotomy to assess whether the laporoscopic approach in endometrial cancer was associated with survival. Our data analyses revealed similar disease-free survival (23 vs. 51, p=0.791) between the two groups. Histology, cervical, and adnexal and serosal involvement were significantly correlated with recurrence in the laparoscopically staged group, whereas CA 125, histology, grade, tumor diameter, cervical involvement, degree of myometrial invasion, adnexal and serosal involvement, and pelvic metastasis were significanly correlated with recurrence in the conventionally managed group. Although the correlation analyses showed some significant correlations that differed between the groups, Cox regression analyses resulted in no association between any of the variables and disease-free survival in either goups. A study by Walker et al.^(8)^ reported short-term outcomes of laparoscopic management of uterine cancer and indicated that the laparoscopic approach was feasable and safe with fewer complications with shorter hospital stay; the study also indicated the necessity for further follow-up to determine the effect of this approach on recurrence and the disease-free survival. A systematic review published in 2013 showed the laparoscopic approach to be beneficial in women with endometrial cancer compared with laparotomy in terms of postoperative complications and length of hospital stay. The authors also directed attention to the requirement for further well-designed randomized clinical trials to assess long-term clinical outcomes, quality of life in particular^(9)^. A review on hysterectomy for endometrial cancer that compared laparoscopy with laparotomy highlighted the growing knowledge, practice, and wider acceptance of the laparoscopic approach by gynecologic surgeons as an alternative surgical method^(10)^. A Cochrane review published in 2012 found evidence to support the role of laparoscopy for the management of early endometrial cancer. The review also suggested that for early-stage primary endometrioid adenocarcinoma of the endometrium, laparoscopy was associated with similar overall and disease-free survival. Laparoscopy was associated with reduced operative morbidity and hospital stay. There was no significant difference in severe post-operative morbidity between the two modalities^(11)^.

Since then, new clinical trials have been published on specific study populations and assessed the safety and effectiveness of laparoscopy in endometrial cancer treatment. One of them, published in 2014, showed that laparoscopy could be considered a safe and effective therapeutic procedure for managing early-stage endometrial cancer in women who were extremely obese with a lower complication rate, lower surgical site infection, and reduced postoperative hospitalization^(12)^. Like previous reports, authors of another study concluded that women with high-grade endometrial cancers staged using minimally invasive techniques experienced fewer complications and similar survival outcomes compared with those staged using laparotomy. The authors also drew attention to the minimized surgical morbidity associated with other comorbidities such as age and requirement for adjuvant therapies^(13)^. A randomized study published in 2013 also indicated the necessity of further studies and concluded laparoscopic surgery to be a safe and reliable alternative to laparotomy in the management of endometrial carcinoma patients, with significantly reduced hospital stay and postoperative complications; however, similar to our results, the authors found that it did not seem to improve overall survival and the 5-year survival rate^(14)^.

Uterine manipulation was blamed for the spread of malignant cells in endometrial cancer during the lapaoroscopic approach; however, a randomized study on this issue showed that insertion of such uterine-manipulating systems did not increase the rate of positive peritoneal cytology or lymphovascular space invasion^(15)^. In our study, the number of resected lymph nodes was significantly higher in the conventionally managed group; however, it is now known that systematic pelvic lymphadenectomy significantly improves surgical staging; it does not improve disease-free or overall survival^(2)^.

There remains a debate on the long-term results of laparoscopic management of endometrial cancer, this study showed similar survival between the two groups.

The laparoscopic approach to endometrial cancer, along with its widely accepted postoperative advantages, has similar disease-free survival but different variables affect reccurrence rates.

## Figures and Tables

**Table 1:**
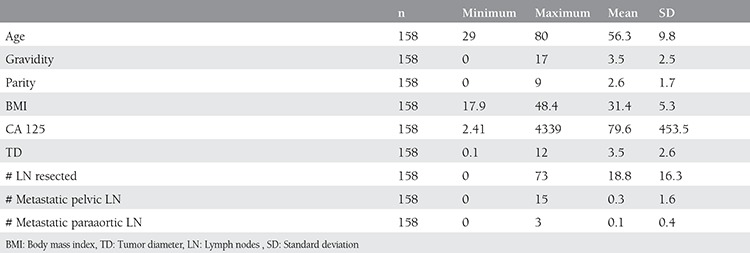
Demographic and the clinical features of the study population

**Table 2:**
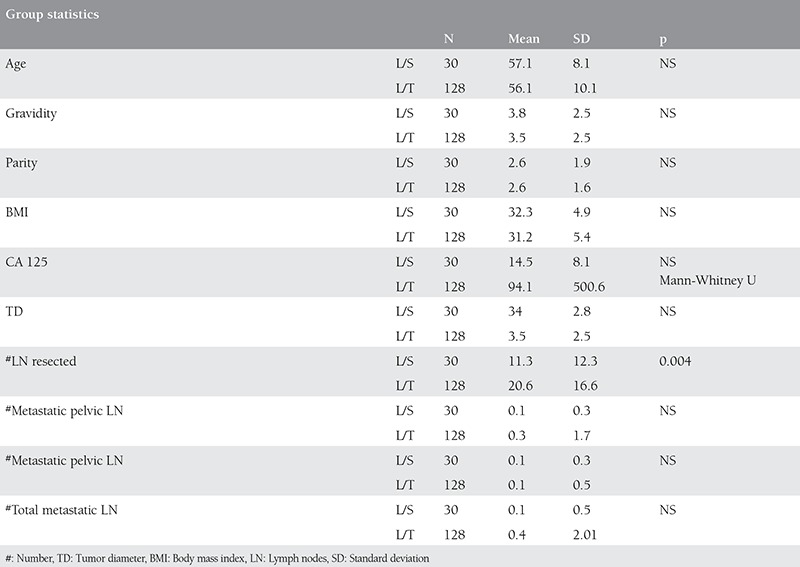
Comparison of clinical and demographic characteristics between the groups

**Table 3:**
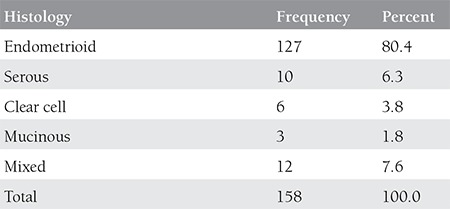
Frequencies of histologic subtypes in the study population
